# Inhibition of IFN-γ-Induced Nitric Oxide Dependent Antimycobacterial Activity by miR-155 and C/EBPβ

**DOI:** 10.3390/ijms17040535

**Published:** 2016-04-08

**Authors:** Yongwei Qin, Qinglan Wang, Youlang Zhou, Yinong Duan, Qian Gao

**Affiliations:** 1 Key Laboratory of Medical Molecular Virology of Ministries of Education and Health, School of Basic Medical Sciences, Fudan University, Shanghai 200032, China; yw_qin@foxmail.com (Y.Q.); wangqinglan2006200@163.com (Q.W.); 2 Department of Pathogen and Immunology, Medical College, Nantong University, 19 Qixiu Road, Nantong 226001, Jiangsu, China; ynduan@gmail.com; 3 Hand Surgery Research Center, Department of Hand Surgery, Affiliated Hospital of Nantong University, 20 Xisi Road, Nantong 226001, Jiangsu, China; youlangzhou@163.com

**Keywords:** microRNA-155, IFN-γ, C/EBPβ, antibacteria, mycobacterium

## Abstract

miR-155 (microRNA-155) is an important non-coding RNA in regulating host crucial biological regulators. However, its regulatory function in mycobacterium infection remains unclear. Our study demonstrates that miR-155 expression is significantly increased in macrophages after *Mycobacterium marinum* (*M.m*) infection. Transfection with anti-miR-155 enhances nitric oxide (NO) synthesis and decreases the mycobacterium burden, and *vice versa*, in interferon γ (IFN-γ) activated macrophages. More importantly, miR-155 can directly bind to the 3′UTR of CCAAT/enhancer binding protein β (C/EBPβ), a positive transcriptional regulator of nitric oxide synthase (NOS2), and regulate C/EBPβ expression negatively. Knockdown of C/EBPβ inhibit the production of nitric oxide synthase and promoted mycobacterium survival. Collectively, these data suggest that *M.m*-induced upregulation of miR-155 downregulated the expression of C/EBPβ, thus decreasing the production of NO and promoting mycobacterium survival, which may provide an insight into the function of miRNA in subverting the host innate immune response by using mycobacterium for its own profit. Understanding how miRNAs partly regulate microbicidal mechanisms may represent an attractive way to control tuberculosis infectious.

## 1. Introduction

Tuberculosis (TB), caused by *Mycobacterium tuberculosis* (*M.tb*), remains one of the world’s biggest health problems. A third of the world’s population has latent TB, with 9.6 million new cases and 1.5 million deaths occurring each year (World Health Organization Global Tuberculosis Report, 2015). The macrophage is a potential target of *M.tb* and is crucial for the pathogenesis of tuberculosis in the early phase of infection [1]. After ingesting the bacteria, macrophages attempt to destroy *M.tb* by a series of antimicrobial mechanisms, including lysosomal degration, antimicrobial peptides, cytokine-mediated mechanisms, reactive oxygen species (or reactive nitrogen species), recruitment of inflammatory immune cells for local inflammatory response, and presentation of antigens to T cells for initiating acquired immunity [2]. However, different layers of negative regulators are harnessed by *M.tb* to efficiently subvert the antibacterial mechanisms of macrophages to promote its proliferation [3].

miRNAs, a class of short non-coding RNAs, reduce gene expression by binding to the 3′-untranslated region (3′UTR) of potential target mRNA or mRNA deadenylation and degradation [4]. miRNAs have been identified as vital regulators of a series of human biological processes, including metabolism, development, differentiation, apoptosis, and cancer [5]. In addition to these valid, established functions in physiological or pathological conditions, miRNAs are increasingly involved in the eukaryotic response to pathogens. Recently, studies have shown miRNA expression in hosts, following infection with exclusively intracellular or extracellular bacteria [6].

miR-155 (microRNA-155) is derived from B-cell Integration Cluster (BIC), a non-coding transcript, and was found to play vital roles in cancer, immune response, and hematopoiesis [7]. miR-155 is induced via Toll-like receptors in macrophages and dendritic cells, and exerts profound effects on the activities of these cells [8,9]. miR-155 promotes the Akt1-mediated regulation of macrophage polarization after *Staphylococcus aureus* pulmonary infection [10]. miR-155 leads to down-regulation of src homology 2 domain-containing inositol-5-phosphatase (SHIP) in monocytes and macrophages following infection with *Francisella. tularensis novicida* [11]. miR-155 has also been found to be involved in the development of Th17 cells, and also during gastrointestinal infection with *Helicobacter pylori* [12,13]. Furthermore, miR-155 is a potential therapeutic target to relieve Th2-medited allergy and anti-helminth immunity [14]. *Mycobacterium bovis* Bacillus Calmette–Guérin (BCG) triggered caspase-dependent apoptosis in infected macrophages due to miR-155 expression [15]. Inhibition of miR-155 hindered the survival of *M.tb* in murine RAW 264.7 and bone marrow-derived macrophages by Bach1 and SHIP1 signaling [16]. miR-155 inhibits IFN-γ-induced autophagy by WNT5A and SHH signaling [17]. In addition, Wang’s results show that miR-155 facilitates autophagy in resting macrophages to reduce endocellular *M.tb* and BCG. These studies indicate that miR-155 may use different bactericidal mechanisms, and plays important roles in extracellular and intracellular bacteria-infected macrophages. However, due to the variety of miR-155 targets and the cross-talk with other signaling pathways, the mechanisms of miR-155 in bacterial infection are very complicated and remain unknown.

The objective of present study is to determine the function of miR-155 in *Mycobacterium marinum* (*M.m*) (a relatively new model organism for the study of mycobacterial pathogenesis [18,19]) infection. We demonstrate that *M.m*-induced upregulation of miR-155 in macrophages downregulated the expression of CCAAT/enhancer binding protein β (C/EBPβ), thus decreasing the production of nitric oxide (NO) and impairing bacterial clearance, which provides a novel subverting bactericidal mechanism of *M.m* by the modulation of microRNA.

## 2. Results

### 2.1. M.m-Mediated Induction of miR-155 (MicroRNA-155) in Macrophage

**Figure 1:**
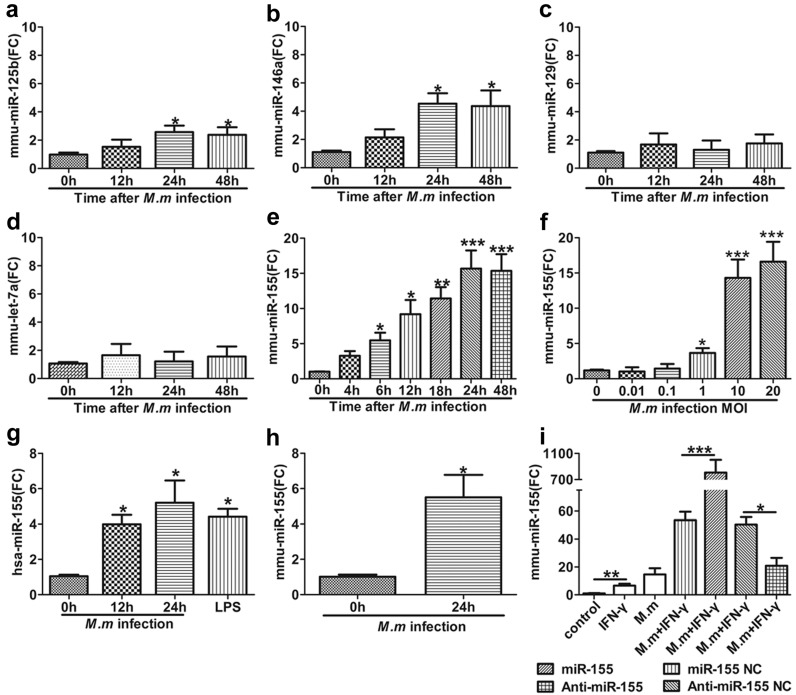
Distinguishing expression of miRNAs in *M.m* infected macrophages. (**a**–**e**) Expression of miR-125b, miR-146a, miR-129, Let-7a and miR-155 in RAW 264.7 cells after *M.m* infection for the indicated times; (**f**) Expression of miR-155 in RAW 264.7 cells at different multiplicities of infection (MOI) for 24 h; (**g**) miR-155 expression levels were detected in THP-1 cells after *M.m* infection or lipopolysaccharide (LPS) (100 ng/mL) treatment; (**h**) miR-155 expression level was examined in mouse peritoneal macrophages (MPMs); (**i**) miR-155 expression was detected in RAW 264.7 cells after transfected with miR-155 Negative Control (NC), miR-155, anti-miR-155 NC and anti-miR-155 prior to infection with *M.m* or treatment with IFN-γ/*M.m* for 24 h. All miRNAs were detected by quantitative real-time polymerase chain reaction (PCRs) (qPCRs). U6 snRNA was used as endogenous control. Experiments were executed with at least three biologically independent replicates, data were shown as the mean ± SEM. * *p* < 0.05, ** *p* < 0.01, *** *p* < 0.001. FC: fold change.

To investigate the role of miRNA in anti-mycobacteria immunity, we selected the high expression of miRNAs in macrophages infected with intracellular bacterial, based on previous reports, such as miR-125b, miR-146a, miR-129, let-7, and miR-155 [8,20,21,22,23]. We firstly detected the expression of those miRNAs in RAW 264.7 cells on *M.m* infection. Among these, miR-146a and miR-125b were moderately upregulated in RAW 264.7 (Figure 1a,b), whereas miR-155 was greatly upregulated. It was also shown that there are time-responsive and dose-dependent inductions. Its induction increased to a peak for ~24 h after *M.m* infection (Figure 1e,f). We obtained similar results in human monocytic THP-1 cells (Figure 1g) and primary mouse peritoneal macrophages (MPM) (Figure 1h). In contrast, miR-129 and let-7a were not shown in detectable upregulation (Figure 1c,d). These results show that miR-155 and miR-146a expression was induced by *M.m* infection.

### 2.2. Effects of miR-155 on Innate Bactericidal Activity

**Figure 2:**
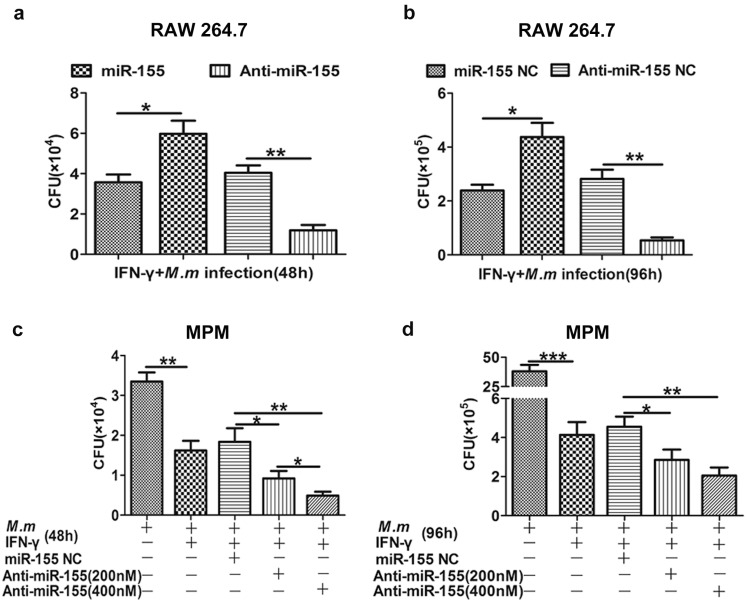
miR-155 negatively regulates the antimycobacterial activity of IFN-γ activated macrophages. RAW 264.7 cells (**a**,**b**) and MPM (**c**,**d**) were transfected with miR-155 NC, miR-155 or anti-miR-155 NC, anti-miR-155 prior to infection with IFN-γ/*M.m* for 48 and 96 h, and intracellular *M.m* survival was quantified by Colony Forming Unit (CFU) assays at the indicated time points. All data represent the mean ± SEM from three independent experiments, * p *<* 0.05, ** p *<* 0.01, *** p *<* 0.001.

The capability of macrophages to kill pathogens is largely intensified by the macrophage activating IFN-γ, which is secreted by activated CD4 T/CD8 T cells and Natural Killer (NK) cells [24,25]. Activation and recruitment of macrophages by IFN-γ increases microbicidal mechanisms [26,27]. The significance of NO in macrophages, on activation by IFN-γ against *M. tuberculosis*, has been well documented, both *in vivo* and *in vitro*, particularly in the murine system [28]. We hypothesized that miR-155 is involved in the bactericidal activity of macrophages activated by IFN-γ. Firstly, we found significant induction or repression of miR-155 expression compared with the corresponding miR-155 Negative Control (NC) (Figure 1i). Moreover, miR-155 exhibited the most significant increase in RAW 264.7 infected with *M.m* combined with IFN-γ. To determine whether miR-155 plays a critical role in antimycobacterial activity, IFN-γ activated RAW 264.7 (Figure 2a,b) or primary MPM (Figure 2c,d) were transfected with miR-155 or anti-miR-155, then infected with *M.m* for two and four days. We found that miR-155 highly promoted the survival of intracellular *M.m* at two and four days. Meanwhile, in anti-miR-155 transfected cells, inhibition of miR-155 boosted the clearance of intracellular bacteria from infected macrophages compared with cells transfected with anti-miR-155 NC at two and four days post infection. In addition, antimycobacterial activity of macrophages was increased, during which anti-miR-155 concentrations change dynamically in MPM.

### 2.3. Role of Nitric Oxide (NO) in miR-155 Promotes the Survival of Intracellular Mycobacteria

**Figure 3:**
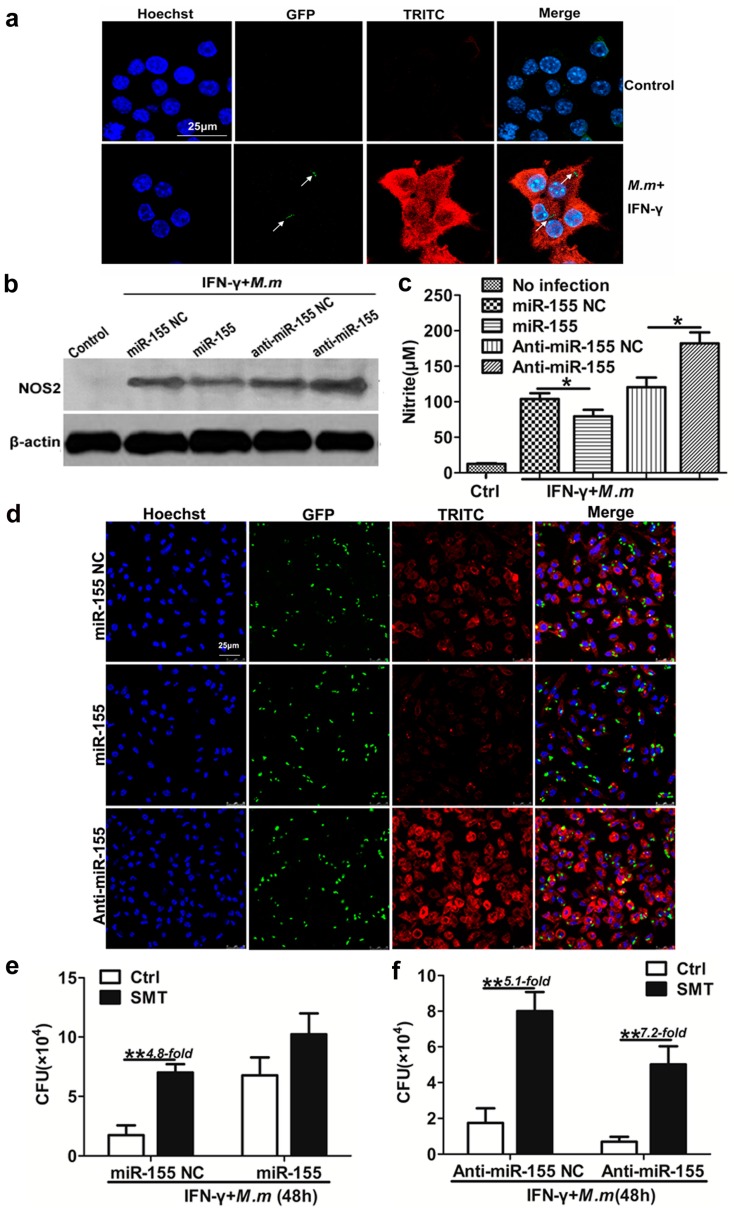
miR-155 promote *M.m* survival in macrophages by inhibition of NO. (**a**) NOS2 expression in the cytoplasm of RAW 264.7 cells. The co-localization of GFP-labeled *M.m* (green) and TRITC-labeled NOS2 (red) in the cytoplasm was examined by confocal microscopy. Arrows indicate *M.m*. Nucleus of RAW 264.7 cells were stained with Hoechst (purple); (**b**) Immunoblot blot analysis of NOS2 in RAW 264.7 cells; (**c**) NO synthesis in RAW 264.7 cell. Cells were transfected with miR-155 NC, miR-155, anti-miR-155 NC and anti-miR-155 followed by IFN-γ treatment and infected with *M.m* for 24 h, supernatant nitrite was determined by the Griess test; (**d**) NOS2 expression in the cytoplasm of MPM cells. Cells were transfected with miR-155 NC, miR-155, anti-miR-155, and infected with GFP-labeled *M.m*/IFN-γ for 24 h. The co-localization of *M.m* (green) and TRITC-labeled NOS2 (red) in the cytoplasm were detected by confocal microscopy. Nucleuses of MPMs were stained with Hoechst (purple); (**e**,**f**) The effect of miR-155, anti-miR-155 and NOS2 inhibitor on *M.m* survival in RAW 264.7 cells. Cells were transfected with miR-155, anti-miR-155 and corresponding NC, pretreated with 1 mM SMT for 2 h, infected with IFN-γ/*M.m* for 48 h. Intracellular *M.m* survival was determined by CFU assay. Data are shown as the means ± SEM of three independent experiments, * *p* < 0.05, ** *p* < 0.01.

The actions of the nitric oxide synthase (NOS2) and the release of NO represent a powerful and necessary antibacterial defense mechanism in host defense against intracellular microorganisms [29,30]. Likewise, NO is absolutely vital in limiting the growth of *M.tb* and, in its scarcity, such as lacking NOS2 in mouse, disease progression is fast and fatal [31,32]. To identify whether the effect of miR-155 on modulating antibacterial activity is exerted through NO, firstly, we detected the cytoplasmic expression of NOS2, which mediated NO production (Figure 3a). To determine the role of NOS2 in miR-155-mediated antimycobacterial activity, IFN-γ activated RAW 264.7 cell or MPM were transfected with miR-155, anti-miR-155, or corresponding NCs, and then infected with *M.m*. We found that NOS2 protein (Figure 3b,d) and supernatant nitrate (Figure 3c) were both increased dramatically by anti-miR-155 transfection (Figure 3b,d). These results indicate that miR-155 could down-regulate expression of NOS2, and therefore NO release.

To further determine whether miR-155 regulates antimycobacterial activity of IFN-γ activated macrophages by affecting NO production, we blocked NO synthase function by using an inhibitor of NO synthase, *S*-Methylisothiourea Sulfate (SMT). Transfection with miR-155 did not significantly affect *M.m* survival, using SMT treated macrophages or not, although the bacterial growth was markedly enhanced when SMT-treated macrophages were transfected with miR-155 NC (Figure 3e). SMT enhanced intracellular bacteria survival in macrophages transfected with anti-miR-155 NC when compared to the control treatment. Transfection with anti-miR-155 drastically enhanced bactericidal activity and reduced intracellular *M.m* survival in macrophages, and the consequences were partly reversed by treatment with SMT (Figure 3f). These results indicate that miR-155 promotes the survival of intracellular mycobacteria by down-regulating NO production, although other mechanisms are also apparently involved in this process. NO and targets of miR-155 function are essential for antimycobacterial activity, and the loss of either weakens antibacterial activity.

### 2.4. Regulation of the CCAAT/Enhancer Binding Protein β (C/EBPβ) by miR-155

**Figure 4:**
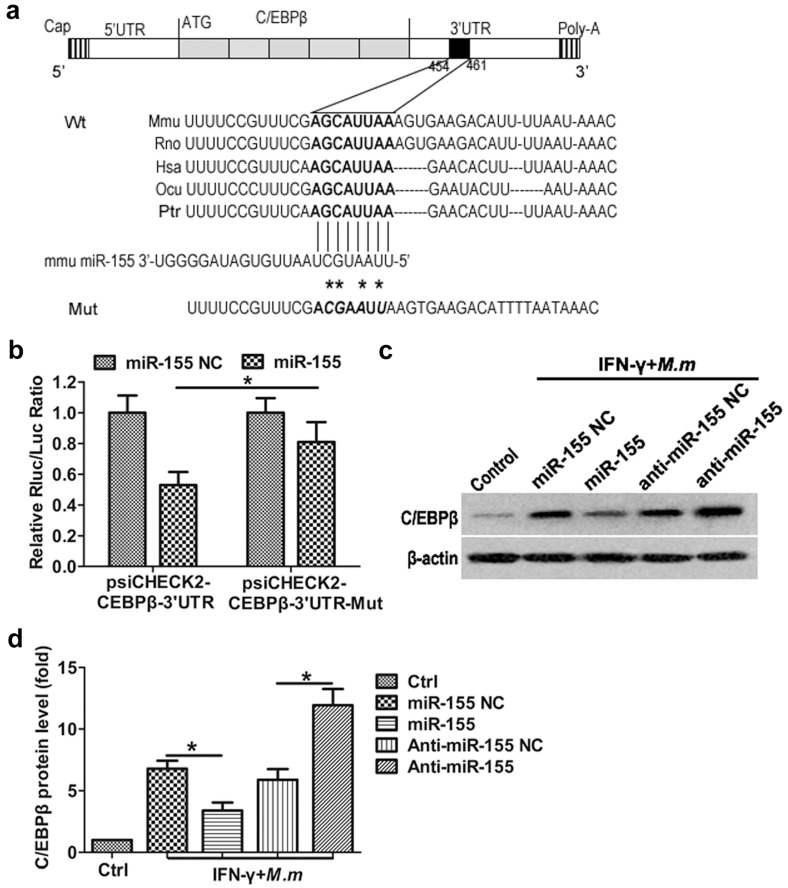
Translational inhibition of C/EBPβ by miR-155. (**a**) Schematic drawing of the C/EBPβ transcript. Sequence alignment and evolutionary conservation between miR-155 and its theoretical binding sites in the 3′UTR of C/EBPβ mRNA from several indicated species, with sequences recognized by miR-155 seed sequence shown in bold. The sequence of C/EBPβ 3′UTR mutant used for reporter assay is also shown (asterisk); (**b**) luciferase reporter constructs containing 3′UTR (psiCHECK2-C/EBPβ 3′UTR) or mutant 3′UTR (psiCHECK2-C/EBPβ 3′UTR-mut) of C/EBPβ gene was cotransfected with miR-155 or miR-155 NC in HEK 293T cell and the luciferase activities (Renilla/firefly) were assayed; (**c**) Western blot analysis of C/EBPβ in RAW 264.7 cells; (**d**) integrated band densities were obtained by scanning using a densitometer. Data are representative of three independent experiments. Results shown are the mean ± SEM. * *p* *<* 0.05.

Computational miRNA target prediction analysis for potential targets of miR-155 with TargetScan showed that miR-155 is able to target the C/EBPβ mRNA 3′UTR, which is greatly conserved in five vertebrate species (Figure 4a). Previous studies have demonstrated that C/EBPβ participates in both inflammatory and metabolic gene regulation [33]. Notably, C/EBPβ is involved in NOS2 gene regulation [34]. It binds to a C/EBPβ motif located at 172 bp of the NOS2 promoter mediates NOS2 expression [35]. Mutation of the corresponding C/EBPβ motif in the murine NOS2 promoter decreases the promoter activity induced by LPS plus IFN-γ in a mouse macrophage cell line J774 [36,37]. Tan found increased expression of C/EBPβ and phosphorylated NF-κB with a concomitant upregulation of NOS2 expression [38]. Gupta’s gel shift assays identically displayed the existence of C/EBPβ among the DNA/protein complexes, which may activate NOS2 gene expression in RAW 264.7 cells [39].

To examine whether miR-155 can repress C/EBPβ expression through direct interaction with 3′UTR, we cloned 3′UTR or 3′UTR-mut of C/EBPβ into psiCHECK2 luciferase reporter plasmid and performed a reporter analysis in HEK 293T cells. Our results showed that cotransfection of miR-155 mimics with C/EBPβ 3′UTR reporter, resulting in inhibition of luciferase activity. However, miR-155 failed to repress the activity of C/EBPβ 3′UTR reporter with a mutated miR-155 seed sequence (Figure 4b). To verify whether transfection of synthetic miR-155 can regulate C/EBPβ expression in IFN-γ activated RAW 264.7 infected with *M.m*, we examined the endogenous C/EBPβ proteins in IFN-γ activated RAW 264.7 infected with *M.m*. Overexpression of miR-155 results in a potent downregulation of C/EBPβ protein levels, whereas concomitant transfection with anti-miR-155 effectively antagonized the miR-155-mediated C/EBPβ inhibition compared to the anti-miR-155 NC (Figure 4c). These data indicate that C/EBPβ is a direct target of miR-155 in IFN-γ activated RAW 264.7 infected with *M.m*.

### 2.5. Role of C/EBPβ in Bactericidal Activities by Macrophages

**Figure 5:**
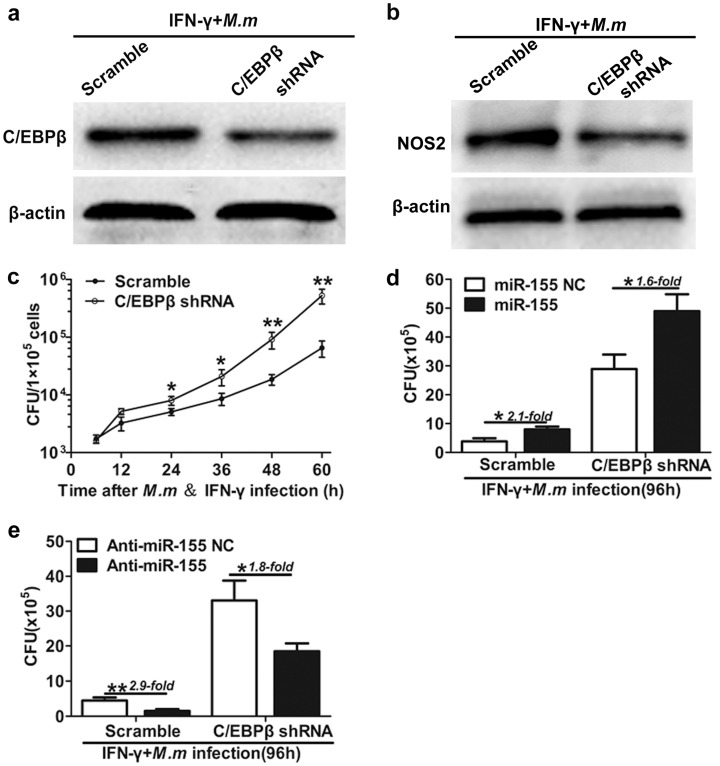
miR-155 promote *M.m* survival in RAW 264.7 cells by inhibition of C/EBPβ and NO. (**a**) Western blot confirmation of shRNA-mediated knockdown of C/EBPβ in RAW 264.7 cell; (**b**) the effect of C/EBPβ on NOS2 expression in RAW 264.7 cells. Cells were infected with either LV-copGFP-scramble or LV-copGFP-shRNA-C/EBPβ. After 48 h, cells were infected with IFN-γ/*M.m* for 24 h and cell lysates were analyzed via immunoblot; (**c**) RAW 264.7 cells infected with either LV-copGFP-scramble or LV-copGFP-shRNA-C/EBPβ were treated with IFN-γ and infected with *M.m* for the indicated times, and intracellular *M.m* survival was quantified by CFU test at the indicated times; (**d**,**e**) RAW 264.7 cells infected with either LV-copGFP-scramble or LV-copGFP-shRNA-C/EBPβ were respectively transfected with miR-155 NC, miR-155 (**d**), anti-miR-155 NC or anti-miR-155 (**e**), followed by infection with IFN-γ/*M.m* for 96 h, and intracellular *M.m* survival was determined by CFU assay. All data are shown as the means ± SEM of three independent experiments, * *p* < 0.05, ** *p* < 0.01.

Reports have suggested that NO is essential for intracellular mycobactericidal activity [31,32]. Previous studies also demonstrated that C/EBPβ participates in NOS2 gene regulation [34,36,37,38,39] and NOS2 exerts potent intracellular mycobactericidal activity [31,32]. To identify whether the effect of miR-155 on modulating antibacterial activity of C/EBPβ is exerted through NOS2, we detected the effects of knockdown of C/EBPβ on antibacterial activities using IFN-γ activated macrophages after being infected with *M.m*. Firstly, RAW 264.7 cells were infected with either copGFP-shRNA-C/EBPβ (C/EBPβ-Knockdown) or copGFP-scramble (C/EBPβ-Wild Type) lentiviral particles. After 48 h, copGFP-expressing RAW 264.7 cells were, respectively, infected with IFN-γ/*M.m* for 20 h. We found that shRNA specific to mouse C/EBPβ significantly inhibited the expression of C/EBPβ (Figure 5a). To explore whether C/EBPβ affects the expression of NOS2 in IFN-γ activated RAW 264.7, we assessed the expression of NOS2 in the absence of C/EBPβ, following *M.m* infection. Lentiviral transduction of C/EBPβ shRNA decreased the levels of NOS2 in IFN-γ activated RAW 264.7 infected with *M.m* (Figure 5b). This indicated that C/EBPβ takes part in NOS2 modulation in IFN-γ activated RAW 264.7 cell infected with *M.m*. Moreover, C/EBPβ markedly exerts antibacterial activity in shRNA-scramble cells compared to knockdown of C/EBPβ cells infected with *M.m* after 24 h (Figure 5c). These data suggested that C/EBPβ is essential for antibacterial activities by IFN-γ activated macrophages infected with *M.m*.

To further investigate the function of anti-miR-155 in regulating the C/EBPβ-induced macrophage bactericidal activity, we performed loss-of-function experiments. We found that miR-155 drastically promoted the survival of intracellular *M.m* in C/EBPβ-KD cells compared with C/EBPβ-WT cells. Similarly, in the C/EBPβ-KD cells, miR-155 significantly increased the survival of intracellular *M.m* compared with the cells transfected with miR-155 NC. Additionally, anti-miR-155 considerably inhibited the survival of intracellular *M.m* in C/EBPβ-WT cells compared with C/EBPβ-KD cells. Meanwhile, in the C/EBPβ-KD cells, anti-miR-155 drastically inhibited the survival of intracellular *M.m* compared with the cells transfected with anti-miR-155 NC (Figure 5d,e). The data from these infection experiments demonstrate the biological relevance of miR-155 in innate host defense: The silencing of miR-155 induction restores C/EBPβ-mediated antimicrobial activity. Taken together, these facts suggest that anti-miR-155 promotes C/EBPβ-mediated antimycobacterial activity, partly by a NO-dependent mechanism in IFN-γ activated macrophages.

## 3. Discussion

**Figure 6:**
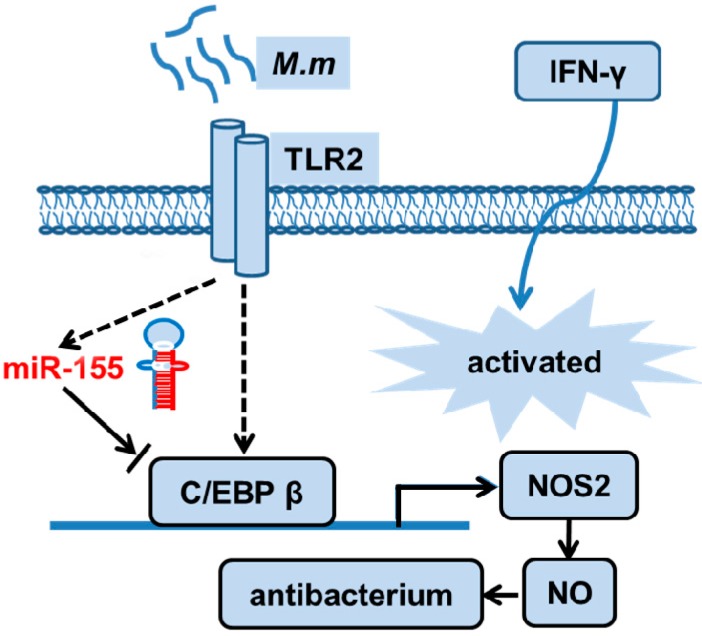
Schematic outline of miR-155-regulated bactericidal activity. miR-155 expression is induced by *M.m* in IFN-γ activated macrophages and mediates translational inhibition of C/EBPβ by targeting its 3′UTR. Successively, C/EBPβ is induced and mediates NO-dependent antibacterial mechanism. Thereby, miR-155 can negatively regulate the NO-dependent antibacterial mechanism by C/EBPβ. Black dash line arrow: direct effect; black solid line arrow: indirect effect; blue arrow: activated.

Here, we report an original function of miR-155 in inhibiting NO production and mycobacterial clearance in IFN-γ activated RAW 264.7 cells by targeting C/EBPβ (Figure 6), which may provide a good understanding of bacteria defense mechanisms against host innate immunity by regulating miRNA.

C/EBPβ was originally identified as a nuclear factor that regulates genes encoding acute phase proteins and cytokines in activated macrophages, such as IL-6, IL-1α, TNF-α, and NOS2 [33,34]. Tanaka’s data show that macrophages from C/EBPβ^(−/−)^ mice have defects in, not only the killing of intracellular pathogens, but also in tumor killing [40]. Consequently, C/EBPβ^(−/−)^ mice are characterized by Lymphoproliferative disorder and imbalanced T-helper response and enhanced susceptibility to *Candida albicans* [41]. These functions of bacteria killing can be elicited as a result of macrophage activation by IFN-γ. Although the mechanism by which IFN-γ activated macrophages kill intracellular pathogens is not completely clear, a series of studies have demonstrated that the generation of NO and reactive nitrogen intermediates play a crucial role in these activities [42]. Macrophage NOS2 is induced by IFN-γ or by nonspecific bacterial components, such as LPS. Interestingly, C/EBPβ-binding sites are identified in the region most important for mediating LPS induction of the mouse macrophage NO synthase gene [43]. Our present study with C/EBPβ shRNA macrophages demonstrated that the production of NO was attenuated in IFN-γ activated macrophages infected with *M.m*.

In addition to anti-miR-155 mediated C/EBPβ-NO antimycobacterial activity, other alternative mechanisms may be implicated in the anti-miR-155 mediated inhibition of bacterial clearance, such as the autophage-dependent mechanism [17]. Wang’s results show that miR-155 promotes autophagy to reduce intracellular *M.tb* and BCG by autophagy [44]. However, Holla reported that miR-155 inhibited IFN-γ-induced autophagy by wingless-type MMTV integration site family member 5A (WNT5A) and sonic hedgehog (SHH) signaling [17]. Additionally, current reported miR-155 functions are involved in apoptosis or autophagy, and little is known regarding the bactericidal activity of NO. Our data demonstrate that miR-155 effectively contributed to the inhibition of NOS2 expression and NO production by targeting C/EBPβ in IFN-γ activated macrophages. These data indicate that miR-155 is implicated in wide signaling cohorts and cross talk with IFN-γ pathways. These mechanisms may act synergistically or in collaboration in mediating antibacterial activities. Alternatively, C/EBPβ may be responsible for the expression of components of a mechanism that enhances NO-mediated antibacterial activity. The results of the bactericidal activity of miR-155 are different to those of Wang and Holla, or our own observations, it is thought to possibly result from a macrophage’s different polarization states, with or without treatment with IFN-γ, which differentially affects their ability to control intracellular pathogens [45]. IFN-γ has been shown to be of crucial value for the killing of intracellular *M.tb* through an autophagy pathway [46,47]. Mice deficient in the IFN-γ receptor are extremely susceptible to pathogens and easily succumb to infection [48]. Importantly, *M.tb*, *M.m* and BCG inducing autophagy, and subsequent bacterial killing by macrophages *ex vivo*, require exogenous treatment with rapamycin, IFN-γ, or starvation [49,50]. In Wang’s experiments [44], it is noteworthy that *M.tb* and BCG-induced autophagy was present in the IFN-γ or rapamycin untreated macrophages transfected with miR-155.

In summary, our study demonstrates that miR-155 suppresses bactericidal activity by targeting C/EBPβ and reducing NO production (Figure 6). These results identify an evasion strategy in which mycobacteria regulate the host miRNA to inhibit the bactericidal response, which may illustrate the potential of using miRNA modulation for the development of a possible preventive and therapeutic route for mycobacteria infection.

## 4. Materials and Methods

### 4.1. Cell Culture and Bacterial Strains

RAW 264.7, HEK 293T and THP-1 cells were grown in Dulbecco’s Modified Eagle Medium (DMEM) or Roswell Park Memorial Institute (RPMI) (Life Technologies, Grand Island, NY, USA) supplemented with 10% fetal calf serum, in a 5% CO_2_ humidified atmosphere at 37 °C. Differentiation of THP-1 cells into macrophages was induced with 10 ng/mL PMA (Sigma, St. Louis, MO, USA) for 24 h. Primary mouse peritoneal macrophages (MPM) were isolated from C57BL/6J mice. Mice were injected intraperitoneal (i.p.) with 2 mL of 4% thioglycollate. Three days later, peritoneal exudate cells were isolated by lavaging the peritoneal cavity with precooling Hank’s Balanced Salt Solution (HBSS). These cells were cultured for 6 h; we used adherent cells as peritoneal macrophages.

RAW 264.7 cell or MPM transfections of 200 or 400 nM miR-155, anti-miR-155 and the respective Negative Control (NC) (Ribobio, Guangzhou, China) were performed according to the manufacturer’s protocols using HiPerFect Transfection Reagent (Qiagen, Valencia, CA, USA). Briefly, the next day, the medium was changed, and RAW 264.7 cells were subjected to *M.m* (multiplicities of infection = 5, MOI = 5) or IFN-γ (200 U/mL)*/M.m*.

*M.m* strain M (ATCC BAA-535) was cultured in Middlebrook 7H9 broth (Difco, Detroit, MI, USA) supplemented with 10% oleic acid-albumin-dextrose-catalase (OADC) (BD Biosciences, San Jose, CA, USA), 0.5% glycerol and 0.05% Tween 80, or on Middlebrook 7H10 (Difco) agar supplemented with 10% OADC and 0.5% glycerol at 32 °C. For infection experiments, *M.m* were grown to logarithmic stage, washed, and diluted in DMEM. The infection of RAW 264.7 cells, THP-1, MPM with *M.m* was performed as described previously [51].

### 4.2. Knock down of C/EBPβ

C/EBPβ shRNA and non-sense shRNA (scramble) (sequences, Table 1) were designed by searching the Sigma online database of validated shRNAs and subcloned between the *BamH* I and *Not* I sites of the pGreenPuro shRNA Expression Lentivector (SBI, Mountain View, CA, USA). Packaging of the lentiviral shRNA expression constructs into pseudoviral particles was performed by transfecting pGreenPuro-shRNA constructs into HEK 293T cells using Lipofectamine 2000 (Life Technologies) together with the packaging plasmids pVSV-G, pRSV-Rev, gag, and TAT (SBI). The virus-containing supernatants were collected, filtered (with 0.45-µm syringe filters), and used to infect the RAW 264.7 cells in complete media containing 10 µg/mL Polybrene (Sigma). At 48 h after lentiviral infection, the stable infected cells were selected with 10 µg/mL puromycin for 24 h.

### 4.3. RNA Isolation and qRT-PCR

Cellular RNAs were extracted using miRNeasy Mini Kit. First-strand cDNAs were synthesized using M-MuLV Reverse Transcriptase (Qiagen). Quantitative PCRs (qPCRs) were performed in a CFX96^TM^ Real Time System (Bio-Rad, Hercules, CA, USA), using SYBR green PCR mixture kits (Takara, Shiga, Japan). Relative quantity of transcripts was calculated using the 2^−ΔΔ*C*t^ formula, as described previously. All PCR primers are reported in Table 1.

### 4.4. Protein Expression

Cells transfected with or without miR-155 NC, miR-155, anti-miR-155 NC and anti-miR-155, followed by IFN-γ treatment and infected with *M.m* for an indicated time; cells were lysed in low stringency lysis buffer, complete with protease inhibitors cocktail. Lysates were resolved in SDS-PAGE gels and transferred onto a PVDF membrane before being immunoblotted with anti-NOS2 (#ab3523, Abcam, Cambridge, MA, USA), anti-C/EBPβ (sc-56637, Santa Cruz, CA, USA) or anti-β-actin (#4970, Cell Signaling, Danvers, MA, USA). Blots were developed by enhanced chemilluminescence (ECL) (Millipore, Molsheim, France).

### 4.5. Immunofluorescence

RAW 264.7 macrophages or MPM were seeded onto coverslips and treated with IFN-γ/GFP-*M.m.* MPM were transfected with miR-155, anti-miR-155 and miR-155 NC, pretreated with IFN-γ/*M.m*. The cells were fixed with 4% paraformaldehyde at room temperature for 30 min followed by cytomembrane permeabilization using 0.1% Triton X-100. Cells were blocked with 5% BSA and incubated with primary (NOS2, CD11b/c) and then second antibodies (anti-rabbit-IgG-TRITC or anti-mouse-IgG-TRITC, Abcam). The coverslips were mounted on a slide with glycerol and cells were viewed using confocal microscopy (Nikon, New York, NY, USA).

### 4.6. 3′UTR Luciferase Reporter Assays

The 3′UTR of mouse C/EBPβ was amplified by PCR from RAW 264.7 cDNA library and cloned downstream of the Renilla luciferase sequence, between the *Xho* I and *Not* I sites of the psiCHECK2 luciferase reporter vector (Promega, Madison, WI, USA). The miR-155 target site in the C/EBPβ 3′UTR was mutated by mutating the 4 nt miR-155 seed match sequence (AGCATTA was mutated to ACGAATT at nucleotide positions 451–458 in the C/EBPβ 3′UTR) using the Site-Directed Mutagenesis kit (Stratagene, La Jolla, CA, USA) according to manufacturer’s protocols. HEK 293T cells described above were cotransfected with 100 ng of psiCHECK2-C/EBPβ 3′UTR or psiCHECK2-C/EBPβ 3′UTR-mut luciferase plasmid, and the indicated miR-155 or NC (final concentration, 50 nM) using Lipofectamine 2000. After 24 h, luciferase activities were detected using the Dual-Glo Luciferase Reporter Assay System (Promega). Data was normalized by dividing Renilla luciferase activity with that of firefly luciferase.

### 4.7. Griess Test

Standards and culture supernatant (50 µL) were, respectively, transferred to 96-well plate, and 50 µL Griess reagent (Promega) was added. After incubation for 5 min at room temperature, the plate was read at 540 nm in the dark and concentrations of nitrate were calculated.

### 4.8. Intracellular Survival Assays

The infected macrophage monolayers were washed thrice with medium and then lysed with 0.1% Triton X-100 (Sigma) to release intracellular mycobacterium. The number of intracellular mycobacterium (CFU) was counted by plating appropriate dilutions onto 7H10 agar plates, as described previously [51].

### 4.9. Statistical Analysis

Differences between two categories were evaluated by two-tailed Student’s *t*-test. Differences between several categories were analyzed using statistical analysis using ANOVA (analysis of variance). When ANOVA detected a significant change, *post hoc* tests were run using the Turkey’s HSD (honest significant difference) test. All data were expressed as mean ± SEM. *p*-values less than 0.05 were regarded as significant.

## 5. Conclusions

In the present study, we identified that C/EBPβ-NO is a direct target for miR-155. Our study elucidates another vital role of miR-155 in NO regulation and mycobacterial survival, which may provide helpful information for developing potential therapeutic tactics and interventions for mycobacteria infection.

## Figures and Tables

**Table 1:** Primers used in this study.

Use	Sequences (5′–3′)
C/EBPβ 3′UTR	Forward: CCGCTCGAGGCCCTGAGTAATCACTTAAAGATGTTC
	Revese: ATAAGAATGCGGCCGCAATGTCTTCACTTTAATGCTCGAAA
C/EBPβ 3′UTR-mut Mutant	Forward: AAGTGAAGACATTGCGGCCGCTGG
	Reverse: AATTCGTCGAAACGGAAAAGGTTCTCAAAATATACA
C/EBPβ shRNA	Forward: gatccCACCCTGCGGAACTTGTTCAATTCAAGAGATTGAACAAGTTCCGCAGGGTGtttttg
	Reverse: aattcaaaaaCACCCTGCGGAACTTGTTCAATCTCTTGAATTGAACAAGTTCCGCAGGGTGg
Scramble shRNA	Forward: GATCCTTCTCCGAACGTGTCACGTTTCAAGAGAACGTGACACGTTCGGAGAATTTTTG
	Reverse: aattcaaaaaTTCTCCGAACGTGTCACGTTCTCTTGAAACGTGACACGTTCGGAGAAg
mmu-miR-155	RT: GTCGTATCCAGTGCAGGGTCCGAGGTATTCGCACTGGATACGACACCCCT
	Forward: CGCCTGTTAATGCTAATTGTGA
	Revese: AGTGCAGGGTCCGAGGTAT
hsa-miR-155	RT: GTCGTATCCAGTGCAGGGTCCGAGGTATTCGCACTGGATACGACACCCCT
	Forward: CGGTTAATGCTAATCGTGATAGG
	Reverse: CAGTGCAGGGTCCGAGGTAT
mmu-miR-146a	RT: GTCGTATCCAGTGCAGGGTCCGAGGTATTCGCACTGGATACGACAACCCA
	Forward: GCCTGAGAACTGAATTCCATG
	Revese: AGTGCAGGGTCCGAGGTAT
mmu-miR-125b	RT: GTCGTATCCAGTGCAGGGTCCGAGGTATTCGCACTGGATACGACTCACAA
	Forward: TCCCTGAGACCCTAACTTGTGAG
	Revese: CAGTGCAGGGTCCGAGGTAT
mmu-miR-129	RT: GTCGTATCCAGTGCAGGGTCCGAGGTATTCGCACTGGATACGACGCAAGC
	Forward: CTTTTTGCGGTCTGGGCTT
	Revese: CAGTGCAGGGTCCGAGGTAT
Let-7	RT: GTCGTATCCAGTGCAGGGTCCGAGGTATTCGCACTGGATACGACAACTAT
	Forward: TGAGGTAGTAGGTTGTATAGTTGTCGTAT
	Revese: CAGTGCAGGGTCCGAGGTAT
RNU6-1	RT: AACGCTTCACGAATTTGCGT
	Forward: CTCGCTTCGGCAGCACA
	Revese: AACGCTTCACGAATTTGCGT

RT: reverse transcription primer of first-strand cDNA synthesis; lowercase letters is restriction enzyme cutting site.
